# Common Problematic Scholarly Activity Project Planning Expectations of Project Novices

**DOI:** 10.51894/001c.21274

**Published:** 2021-04-13

**Authors:** Samuel J. Wisniewski, William D. Corser

**Affiliations:** 1 College of Osteopathic Medicine Statewide Campus System Michigan State University

**Keywords:** quality improvement, research, project novice, graduate medical education, learner expectations, project planning, scholarly activity

## Abstract

**INTRODUCTION:**

Scholarly Activity (SA) projects, whether using methods more traditionally associated with research and or “quality improvement” projects, have been shown to confer value to resident physicians and other project novices in multiple ways. The inclusion of community and university-based residents and faculty in spearheading SA projects has led to improved understanding of medical literature and enhanced clinical practices, arguably producing more “well-rounded” physicians.

**PURPOSE OF PAPER:**

The primary purpose of this paper is to provide a summary of problematic expectations frequently assumed by project novices when developing and conducting SA projects.

**RESULTS:**

The authors will discuss a total of 26 problematic project-related novice expectations during five typical project phase categories.

**CONCLUSIONS:**

Learning to navigate the complexities of training to become a practicing physician, while also planning high quality SA project designs has been and will continue to be a complex challenge. The authors hope that this article can be used by supervising faculty and other graduate medical education mentors to assist the SA project novice (SAPN) plan SA projects. By establishing realistic expectations during project planning phases, the SAPN can avoid potential missteps that typically impede SA project completion.

## INTRODUCTION

Scholarly Activity (SA) projects, whether using methods more traditionally associated with research and or “quality improvement” (QI) projects, have been shown to confer value to residents and other SA project novices in multiple ways.^1–6^ First, the conducting of SA projects has been associated with increased prospective fellowship opportunities.^1–3^ Second, the inclusion of community and university-based residents and faculty in SA projects leads to improved understanding of the medical literature and enhanced clinical practices, arguably producing more “well-rounded” physicians.^4–6^ Finally, while conducting SA projects during a residency can be quite complex,^7–11^ resident physicians, with their “new set of eyes” perspective, may in some ways be in a better position to conduct SA projects to investigate patient care inconsistencies compared to their clinically experienced faculty.^10,12–14^

The authors’ Statewide Campus System (SCS) in the Michigan State University College of Osteopathic Medicine (MSUCOM) is a consortium of graduate medical residency programs across the state of Michigan.^15^ Since 2015, the authors have assisted hundreds of residents and faculty in design and completion of community-based SA projects.^16–19^ They have completed over 2,500 total consultation hours to date on phases of over 450 SA projects in more than 10 clinical specialties.

The authors’ consultations have included developing initial project description statements (PDS), refining institutional review board (IRB) applications, conducting statistical analyses and copy-editing of dissemination products.^16,18^ Their SA experiences have equipped the authors^20^ with an appreciation of the varied challenges typically faced by the SA project novice (SAPN).

### Purpose of Paper

The primary purpose of this paper is to provide a summary set of problematic expectations frequently encountered when working with the SAPN in the development and conducting of SA projects. The intended audiences of this paper are resident physicians and their mentors (e.g., chief residents, residency program officials, clinically experienced colleagues) who develop and implement SA projects across the nation.^11,14,21–29^ The authors will discuss a total of 26 problematic SAPN project planning expectations in five categories. (Table 1) To guide readers, these expectations are listed in Table 1 in typical chronological order of SA project developmental phases. These expectations are presented throughout as “bulleted” quotations summarily paraphrasing varied but related comments the authors have often received in relation to these expectations across >2,500 hrs of consultation time.

**Table 1.** 26 Problematic Project Planning Expectations of Scholarly Activity Project Novices

**I. Initial Project Design**
• “My project must be an entirely new project design.”
• “My “idea” will lead me to the best project design.”
• “Writing out my project activities is less important.”
• “I will develop this project by myself.”
• “Quality Improvement projects are less valuable.”
• “Replication projects will be less publishable.”
• “Scholarly activity projects are either QI or research.”
• “Projects with pre-existing data will be easier.”
• “I’m a clinician so I should really intervene somehow.”
• “The “theoretical” stuff is less relevant.”
• “My analyst will help overcome any project limitations.”
• “Statistically significant results are needed for a nice publication.”
• “A larger sample will compensate for other methodological shortcomings.”
• “My clinical abilities will dictate most project elements.”
• “Great results can refashion knowledge in my clinical specialty.”
**II. Institutional Review Board Application**
• “The IRB will probably impede my work.”
**III. Data Collection and Project Management**
• “My team members will manage their own project assignments.”
• “Formal project timelines do not have much value.”
**IV. Data Set Preparation and Analysis**
• “My sample will just have to provide me with enough statistical power.”
• “My selection of measures can come later.”
• “Extraneous influences don’t really interest me.”
• “My analyst will send me my results in a few days.”
• “Statistical significance (i.e., p-values) is what matters.”
• “If I don’t produce the results I expected, then my analyst can work my data until we find something!”
**V. Dissemination of Results**
• “Generating conclusions from my results should be a relatively simple task.”
• “Numerous poster presentations are about as good as a publication.”

## I. Initial Project Design

- ***“My project must be an entirely new design.”***

The concept of building on existing work (e.g., replication or follow-up projects) is less often recognized by the SAPN. This area of graduate medical education (GME) project scholarship has been investigated during resident survey projects concerning perceived value by the SAPN of SA project types.^3,9,13,21^ Conclusions were that many SAPNs may be less familiar with the value of replicating published project articles, possibly due to pressure towards producing SA projects from more “established” (i.e., more readily recognizable) types of studies, e.g., randomized clinical trials (RCTs), etc.^25,26,29–31^

- ***“My”idea" will lead me to the best project design."***

The SAPN may frequently start with nebulous project ideas they expect to somehow yield feasible SA project designs.^9,10,32^ For example, the authors have heard from the SAPN, *“I thought it would be interesting to study individuals with condition x.”* Although this type of orientation may eventually lead to useful descriptive *pilot* (i.e., more exploratory, descriptive) projects,^5,31,33^ the authors have generally concluded that this may comprise an unrealistic desire to study especially terribly complex healthcare topics in initial projects.

Ideally, the SAPN can be first steered toward appealing topics related to their compelling clinical experiences.^21,31,32^ A well-written project description statement (PDS) will describe overall project aims and specific project questions, sampling and data collection plans and prospective post-project ways to disseminate final results.^29,32^ Two examples of actionable SA project questions include: a) *“What office visit patient characteristics (age, sex, etc.) are associated with hypertension management outcomes at our clinic?,”* or b) *“What kinds of educational materials (physical or virtual, etc.) do our patients prefer after they are diagnosed with Type 2 diabetes?”*

- ***“Writing out my project timeline is less important.”***

The SAPN may underestimate the need to write out project timeline elements (e.g., planning when to access/collect patient data, submit IRB application, have data analyzed, etc.) when organizing their initial project design.^25,28^ Refining one’s timeline with mentors may be viewed as either unnecessary or too time-consuming, sometimes perhaps based on a naive expectation that “everything will come together in the end.”^5,10,33^ However, the authors and others have regularly emphasized to the SAPN that a detailed timeline can facilitate: a) successful project completion, and b) reduce the time needed for institutional review board (IRB) project approval, as well as conduction of analyses and interpretation of final results.^4,5,13,18^

- ***“I will develop this project by myself.”***

This approach may come from a simplistic notion that the SAPN can be capable of autonomously completing their SA projects (i.e., “do all the work.”). The authors’ experiences to date have frequently led them to recommend that the SAPN undertake projects with at least one or more colleagues.^16,18^ It will almost always be advantageous to have “additional sets of eyes” throughout not only the initial project design and PDS, but also in subsequent steps of developing IRB application drafts, data collection sheets, drafts write-ups, etc.^2,5,24,25^

- ***“Quality Improvement projects are less valuable.”***

This misunderstanding has come from medical school curricula oftentimes placing a higher value on randomized controlled trial or other complex designs, generally signaling that QI projects are “not as rigorous.”^21,28,34^ However, it has been emphasized in GME literature that QI project results can be more often rapidly incorporated into patient care processes and facilitate an institutional “collective learning” in healthier GME environments.^16,18,19,21,24,33^

- ***“Replication projects will be less publishable.”***

This appears to come from a naive assumption that SA projects need to be terribly novel and “stand out” from earlier works to yield much value. However, experts have asserted that innovative replication projects (i.e., using different samples, settings, etc.) can enhance the “external generalizability” of earlier project findings and conclusions to other settings.^9,27,32,33^

- ***“Scholarly activity projects are either quality improvement or research.”***

Although QI and research are common distinctions for SA projects many projects can contain elements of both design types.^13,16,18,22^ For example, the results of survey questionnaire pilot projects or projects evaluating patient interventions may simultaneously provide generalizable “research” knowledge to other settings at the same time they provide insights into potential individual healthcare settings (i.e., more QI) improvements.^6,22,28,29^

- ***“Projects with pre-existing data will be easier.”***

Although SA projects that utilize pre-existing or secondary data (e.g., already collected electronic health record data) *can* first appear to be *relatively* straightforward, the methodological and analytic complexities involved in examining multi-faceted healthcare phenomena can still be considerable.^18,27,35^ In fact, just as with studies collecting “new” (i.e., prospective) data, a well-developed PDS for a project using retrospective data will just as likely include additional project measures (i.e., “exposures” and “outcomes” of interest) to study complex medical topics. This can compound the scope of project question(s), suitable project measures, and number of hypothesized relationships for projects using pre-existing data as well.^29,31,35^

For example, if a hypothetical project used pre-existing data to investigate the prevalence and incidence of Alzheimer’s dementia during a specified time period, the SAPN could experience substantial difficulty confirming when and how the diagnostic criteria for Alzheimer’s have changed. In addition, comparing different forms of diagnosed Alzheimer’s remains difficult due to the ongoing lack of understanding exact causal mechanisms.^36^ In many instances, extracting such understandings from pre-existing data may be especially difficult compared to using prospectively collected, controlled samples.

- ***“I’m a clinician so I should really intervene somehow.”***

The inherent desire of clinicians to actively intervene with patients during SA projects (e.g., develop some sort of randomized clinical trial or pre-post-intervention project) is quite understandable. However, GME authors have frequently observed uncompleted SA projects due to the complexities involving the numerous steps required to control, dose out and deliver complicated patient interventions.^9,10,18,22,28^

This inherent preference usually requires tighter patient/intervention “controls” than more exploratory SA projects with more flexible project periods, broader sampling plans, etc.

- ***“The”theoretical" stuff is less relevant."***

An inadequate understanding of the theoretical or conceptual dimensions of a SA project topic may come from: a) only considering the conceptual dimensions of more “interesting” clinically compelling variables, or b) not considering additional possible confounding (i.e., factors that are both related to an “exposure” and “outcome” of interest) influences.^3,5,18,30,31^ A SAPN can end up with ambiguous or incomplete results from failing to consider the theoretical/conceptual assumptions underpinning their project topic.^27,31^ Again, this is an important reason to ensure that your SA project topic has been thoroughly researched (i.e., literature review) early on, so that the SAPN can delineate all relevant covariate and potential confounding variables.

- ***“My analyst will help overcome any project limitations.”***

During project designs, the authors have observed the SAPN sometimes failing to consider the “bigger picture” (i.e., entire project scope, how project design elements (should) fit together). This narrow orientation can lead to later blatant data “trolling,” or “dredging” requests of analysts, perhaps an analytic “tail-chase” (i.e., repeatedly trying to adjust project data based on unclear SA project questions/objectives).^35,37^

Failing to thoughtfully consider how one’s results could be utilized in contemporary healthcare settings during initial project design discussions can also lead to the “So what does all of this mean?” question the authors have heard from the SAPN after “final” analyses.

Ideally, the final conclusions stemming from SA project results will be derived from a concise PDS and well-specified analytic methods and measures identified early in the project timeline.^11,27,30,31,38^

- ***“Statistically significant results are needed for a nice publication.”***

One misguided conclusion regarding statistical significance (i.e., a coefficient Alpha p-value of less than 0.05) is that, *"achieving statistical significance is what really matters."*^39^ Statistically significant results from a SA project are generally more valuable when the core components of a project design (e.g., establishment of primary aims, hypotheses, etc.) have been thoughtfully specified to make project results more credible or translatable into practice.^6,37,38^

- ***“A larger sample will compensate for other methodological shortcomings.”***

This misunderstanding is related to a “The larger the sample, the better” fallacy. A SAPN may presume, “If I have a larger sample, I will more likely find some interesting (i.e., publishable) results.” However, having a “large-enough” sample size may still prevent a project team from drawing meaningful conclusions. The SAPN must ensure methodological design elements reflect the existing science (i.e., background literature) in the topic area and have been adequately (i.e., well methodologically) planned out.^29,35,38^

- ***“My clinical abilities will dictate most project elements.”***

Well educated SAPN physicians may be tempted to overestimate the extent to which their clinical knowledge and aptitude will be readily transferred into SA project designs.^1,3,5,6^ However, experienced physicians have recognized that skills required for most SA projects will be fundamentally different than their “clinical skills.”^2,14,18,22^

In fact, more experienced clinicians have noted that this difference (i.e., the terminology/methodology variations between a MD/DO (clinical) context to that of a PhD (research)) could be compared to learning an entirely different language.

- ***“Great results can refashion knowledge in my clinical specialty.”***

This can be related to most project leaders’ tendency to “go big” when envisioning prospective projects.^3,4,6^ Both residents and faculty may also have an understandable desire for their SA projects to “stand out” among colleagues.^2,3,14,24^ Although the scope (e.g., sample sizes) of any subsequent projects may feasibly increase, starting with too grandiose a SA project plan can frequently end up being both frustrating and self-defeating.^18,19,23,28^

### Take Home Points for Initial Project Designs

In summary, when considering the first phase of SA project planning, development of the initial project design, the authors recommend:

As much as possible, avoid:

- Starting too “big”
  - The complexities of SA project development are only exacerbated by attempting to tackle more advanced project designs (e.g., RCTs, longitudinal cohort studies of complex conditions).
  - Instead, the authors advise the SAPN to consider starting by either:
    - Getting involved with an ongoing project headed by a senior resident or faculty, which can allow you to less painfully “dip your toes” into the SA project realm, or,
    - Start “smaller” by developing more of a “pilot” project on which future work by you and/or others can build upon.
- Trying to reinvent the wheel
  - Recognize the value of replication studies as well as “borrowing” design elements from completed studies.
- Taking on a SA project all on your own
  - A SA project team can not only help shoulder the burden (i.e., time and effort) of completing/implementing various project tasks, but can also offer critical “new sets of eyes” to developing project plans, protocols, etc.
- Ignoring the value of establishing (and sticking to) project timelines
  - The complexities inherent to many SA projects will necessitate managing a multitude of moving parts. As SAPN (and project lead), establishing and maintaining project timelines (and adjusting them when necessary) is, from the authors’ experience, essential to mitigating avoidable mid and late-stream project issues that can arise.
- Having unrealistic expectations for data analyses
  - First, a statistically significant (i.e., p-value less than 0.05) result will not matter much if the primary project questions (i.e., aims) of your SA project are not clearly defined.
  - Further, assuming that if you collect a “big enough” sample for analyses that this will mitigate inherent SA project design limitations (e.g., a large sample for a poorly defined and or redundant project aims) is inadvisable.

## II. Institutional Review Board (IRB) Application

- ***“The IRB will probably impede my work.”***

Understandably the SAPN may perceive their first IRB applications and the review process to be needlessly onerous and lengthy.^5,6,18,30^ However, this perception can come from an incomplete understanding of what IRBs do to protect human subjects. Additionally, SA project applications may unfortunately be subject to seemingly “inconsistent” decisions and ambiguities during IRB reviews.^3,6,13,18^ The authors of this paper and others have also found that the SAPN may underestimate how lengthy IRB review processes could be.^10,11,16,31^ In most cases however, additional value can be conferred as IRB project application reviews can serve as a “double check” to further improve or clarify project design elements still relatively early on.^2,5,16^

### Take Home Points for IRB Application Considerations

- The IRB process is necessary to ensure patient safety/confidentiality while working on any type of SA project. This responsibility lies not only with the IRB, but with all SA project team members. Reorienting ones’ perspective to appreciate the service IRBs provide in making certain that those patients taking part in your study have their rights as persons respected is pivotal. It may also be helpful to think of the IRB as an extra “new set of eyes” to help you answer integral questions about your study design relatively early on.

## III. Data Collection and Project Management

- ***“My team members will manage their own project assignments.”***

Whether or not they are listed as Principal Investigator (PI) of record, a SAPN will often still be responsible for the supervision of most SA project activities. Forming a project team and developing processes/procedures agree upon by the team can help the SAPN “divide up the work,” increasing the likelihood that tasks will be completed in a timely and thorough manner.^1,4,29,31^

- ***“Formal project timelines do not have much value.”***

There may be two salient factors associated with this problematic conclusion. First, the SAPN may have an unrealistic sense of how long the completion of many activities and phases of SA projects may actually take.^5,9,13,14^ Secondly, the authors and others have observed the SAPN struggling with meeting project-related demands when juggling other competing non-project role demands.^9,10,19,24,25,27^ Project planning timelines/deadlines have been shown to help the SAPN complete project demands and later posters, presentations and manuscripts.^18,24,27,29,31^

### Take Home Points for Data Collection and Project Management

- The onus is on the project lead (SAPN) to keep the project on track. The data collection phase is arguably one of the more important instances when project management is particularly relevant due to often involved processes regarding accessing, organizing, and cleaning data.
- Consider two types of data collection.
  - The first is for retrospective data. In some instances, this process may be fairly straightforward, e.g., if there is a dedicated “data” individual at your health system who can pull EHR data for you. Even then however, you or a team member may be tasked with going through mass amounts of data to extract/format those primary variables of interest.
  - In the case of prospective data collection, there are a myriad of considerations. Whether you are collecting data more directly (as with surveys administered in a health system environment, etc.) or indirectly (perhaps you have an EHR that automatically already pulls/records the primary variable data of interest), time will also be a significant consideration. For example, in the direct case, you have to account for how much additional time these surveys will take up for a patient visit. If the survey is too long the patient may opt out or choose not to finish. In the case of “indirect” data, time is also relevant in that you have to consider how long it might take for the health system or community-based practice to collect a sufficient number of data points to meet your minimum sample size requirements.

## IV. Data Preparation/Cleaning/Analysis

- ***“My sample will just have to provide me with enough statistical power.”***

Although this expectation may be forced by the circumstances of what size sample might be available or how long a project period is feasible (i.e., within the limits of remaining residency, or event submission deadlines, etc.), the issue of minimal sample size to obtain an adequate level of “statistical power” should be considered early and with your data analyst during initial project design discussions.^35,37–39^ There are currently several free online minimal sample size software calculators available, such as *G Power 3.1.9.4*.^40^

- ***“My selection of measures can come later.”***

The early project measures that need to be selected by the SAPN will be heavily influenced by their selected project topic and approach.^23,27,35,41^ As witnessed by the authors, downplaying the importance of selecting one’s project measures can a) increase the chances that a project loses “focus,” and b) ultimately skew subsequent data analyses and results interpretation.^41–43^ As SA analysts, the authors continue to receive data sets with unclear variables and ambiguous data set codings that prompt us to ask the SAPN, “So, what were the primary relationships you were trying to examine?” or “What prompted you to choose this topic in the first place?” Clearly defining project measures *early on* increases the likelihood of being able to successfully submit any sort of dissemination products, e.g., project write-up manuscript, etc.

- ***“Extraneous influences don’t really interest me.”***

This problematic assumption relates to not appreciating the possible importance of extraneous/confounding factors, (i.e., those factors that may influence or “skew”) the SAPN selected “outcome(s) of interest.”^42,43^ Consideration of these possibilities during early project planning can significantly improve a SAPNs’ subsequent ability to draw credible conclusions from their final results.^41,42^

- ***“My analyst will send me my results in a few days.”***

This unrealistic expectation may develop when mutual project-related expectations are not established between the SAPN and their data analyst.^16,35^ Both the SAPN and analyst should agree on respective deadlines and capacities, continually updating one another when adjustments are required.

- ***“Statistical significance (p-values) are what matter.”***

Generally, receiving one or more “statistically significant” p-values from one’s analyses are viewed as desirable."^37,39^ However, poorly designed projects may be capable of generating significant p value levels without really contributing much to medical knowledge or practice.^38,39^

Alternatively, considering the “effect size”^38,39^ (i.e., describing your results in terms of magnitude to affect treated groups) or “clinical significance,” (i.e., evaluating whether your results can make a difference in patient care processes) may be more appropriate.^44^ In addition, well-designed projects that fail to attain statistically significant (i.e., “negative”) findings can still help inform how future project designs are planned or what measures may need to be better indicated.^2,5,13,38,44^

- ***“If I don’t get the results I expected, then my analyst can work with my data until we find something!”***

This potential issue is related to what was briefly discussed before regarding data “fishing” or “dredging” and disregarding the value in explicitly defining project measures and theoretical relationships during project development.^29,35,37^ The initial project design process should include clearly delineated study questions that your SA project is designed to answer. Expected outcomes, also known as hypotheses, are tested and are either accepted or rejected. Any additional relationships that may come to mind (e.g., unconsidered possible effects of demographics variables such as age or gender, other relevant variables, etc.) over the course of conducting your project should at least briefly be discussed in a “limitations” section of your write-up.

### Take Home Points for Data Preparation/Cleaning/Analysis

- The importance stressed on establishing your primary variables and relationships between those primary variables of interest (i.e., SA project aims) from earlier in this paper particularly comes into play during this project phase. Getting to this point of the SA project process and not having a clear picture of what your SA project aims are is perhaps one of the most common issues the authors experience. As such, the authors cannot emphasize enough recommending the SAPN avoids waiting until this later point to tackle these very important SA study questions. Contact your analyst very early on (i.e., not months, not weeks, and never days before a deadline).
- Consider that SA project consultants may have multiple competing demands (other SA consult/analytic requests, etc.) that come with impending deadlines that can easily occupy them for up to 2-3 weeks at a time. This underscores the importance of establishing communication transparency as early as possible (ideally in the initial design stage) as well as maintaining communication on any and all deadlines changes that may occur.

## V. Dissemination of Results

- ***“Generating conclusions from my results should be a relatively simple task.”***

The SAPN may view the task of interpreting their project results as a binary process of whether they can find what they were expecting to find. At a minimum, a SAPN should specifically ask a) whether their pre-formulated project hypotheses were supported, b) whether their results match previous study findings, and c) how their findings might contribute to future projects in the area. The notion that a SAPN may possibly conclude their project with “more questions than answers” should be considered and perhaps, expected.^3,5,14,32^

- ***“Numerous poster presentations are about as good as a publication.”***

The extent to which a publication may be weighted relative to poster or podium presentations has varied from accreditation reviewers.^7,11^ It is still safe for the SAPN to conclude that presenting one or more poster presentations will not usually be considered equivalent to publication of a SA paper, particularly those that are nationally indexed (e g., PubMed).^45^ In most scenarios, publishing a SA project manuscript (i.e., as opposed to case report/series papers) is viewed more positively.^4,6,29^

### Take Home Points for Dissemination of Results

- Avoid deviating from responding to your primary aims. Discuss what you set out to examine (your primary aims) and whether your results corroborate your hypotheses or not. Next, discuss why the results did or did not line up with what you originally posited would happen, tying back into your introduction/background literature. In other words, examine the ways in which your SA project results agree or disagree with other literature on your topic area and explore why (e.g., fundamentally different patient populations in terms of demographics or occurrence of conditions, etc.) these similarities or differences could exist.
- Expand on the limitations of your study and how future studies could refine the methods of your SA project to further investigate your topic of interest.

## DISCUSSION

The number of articles concerning this area of GME scholarship is still quite limited. Learning to navigate the complexities of training to become a practicing physician, while also planning quality SA project designs will very likely continue to be a complex challenge.^2–5,9,12^

To establish a mutually beneficial relationship with your team members and increase the likelihood that you will complete your project, the authors recommend that SAPN prioritize the following.

### For project overall:

- Start early and consider topics that may interest you from your medical student/resident experiences. For example, are there particular points of care (patient hand offs, error-reporting, etc.) you have noticed that seem like they could be improved? If this has not already been examined, look into where and how (EHR data person at health system, etc.) you might obtain relevant data. Alternatively, perhaps someone at your system has previously examined the base data? If so, you could develop a follow-up study to examine the issue in further detail (e.g., if the previous study found that proper procedures for patient hand offs were only occurring 50% of the time and you want to investigate how to increase compliance).

### For IRB considerations:

- Recall that the IRB is a necessary and extremely important part of the SA project process to ensure patient safety and confidentiality. Always be polite/courteous and address all IRB-related requests in a timely manner.

### For data analyst/project consultant:

- Establish who your data analyst will be early on. This is extremely important as the analyst can assist you in working through selecting your measures, interpreting the existing literature, identifying primary variables/relationships, constructing a suitable data collection sheet, calculating SA project minimal sample size, etc. for your initial project design.
- Establish mutual timelines, i.e., letting the analyst know if you as the SAPN have an upcoming abstract or other submission (manuscript) deadlines well in advance. This can allow the analyst to better coordinate/be able to assist you in meeting your important deadlines.

The authors hope that this article can be used to assist the SAPN and their mentors plan SA projects with realistic expectations during project planning phases, to avoid potential missteps that could impede project completion. This overall challenge is likely to expand since current ACGME accreditation standard trends certainly appear to be moving toward increased SA project and dissemination product requirements.^6,8,11,16^

## CONCLUSIONS

Most SAPN will perceive increasing SA project standards as simultaneously challenging and onerous.^5,9,18,21,23^ Ideally, the SAPN in most GME settings can be effectively guided by experienced mentors to produce feasible project plans that generate useful results for subsequent projects.^2,4,22,24^

Future studies from both community-based and tertiary or university based hospitals to investigate the occurrence of such SAPN project-related misunderstandings will be required to more fully understand the perspectives of SA clinicians in our nation’s many healthcare settings.

### Conflicts of Interest

The authors declare no conflicts of interest, financial or otherwise.
